# Development of a wastewater based infectious disease surveillance research system in South Korea

**DOI:** 10.1038/s41598-024-76614-4

**Published:** 2024-10-19

**Authors:** Yun-Tae Kim, Kyungwon Lee, Hyukmin Lee, Bokyung Son, Myeongwon Song, Seung-Hyun Lee, Miran Kwon, Dong-Soo Kim, Tae-Hun Noh, Sanghoo Lee, Young-Jin Kim, Mi-Kyeong Lee, Kyoung-Ryul Lee

**Affiliations:** 1 Department of R&D Innovation Center, Seoul Clinical Laboratories, Yongin-si, Gyeonggi-do Republic of Korea; 2 Department of Laboratory Medicine, Research Institute of Bacterial Resistance, Yonsei University College of Medicine, Seoul, Republic of Korea; 3 Seoul Clinical Laboratories, Yongin-si, Gyeonggi-do Republic of Korea; 4 SCL Healthcare Inc. Gyeonggi-do, Yongin-si, Gyeonggi-do Republic of Korea; 5 Department of R&D Innovation Center, Seoul Clinical Laboratories, Yongin-si, Gyeonggi-do Republic of Korea

**Keywords:** Wastewater-based epidemiology, Respiratory viruses, Pneumonia-causing bacteria, Diarrhea-causing microorganisms, Real-time PCR, Public health, Pathogens, Policy and public health in microbiology, Microbiology, Water microbiology, Epidemiology

## Abstract

Wastewater-based epidemiology has been used in pathogen surveillance for microorganisms at the community level. This study was conducted to determine the occurrence and trends of infectious pathogens in sewage from Yongin city and the relationships between these pathogens and the incidence of infectious diseases in the community. From December 2022 to November 2023, we collected inflow water from six wastewater treatment plants in Yongin city twice a month. The analyzed microorganisms included 15 respiratory viruses, 7 pneumonia-causing bacteria, 19 acute diarrhea-causing pathogens, SARS-CoV-2, Zika virus, hepatitis A virus, poliovirus, Mpox, and measles. They were detected through real-time PCR and conventional PCR. The concentrations of 9 pathogens among them were additionally analyzed using quantitative real time PCR. The correlation was confirmed through statistical analysis with the rate of detection for pathogens reported by the Korea Disease Control and Prevention Agency. Influenza A virus, human adenovirus, and human rhinovirus were moderately correlated (rho values of 0.45 to 0.58). *Campylobacter* spp. and sapovirus were strong correlated (rho values of 0.62, 0.63). Enteropathogenic *E. coli*, human coronavirus, and norovirus GII were very strong correlated (rho values of 0.86 to 0.92). We were able to identify the prevalence of respiratory viral infections, pneumonia, and acute diarrhea-causing pathogens in the community through wastewater-based epidemiology data. This study will be helpful in establishing a system for future surveillance of infectious diseases present in sewage.

**Supplementary Information:**

The online version contains supplementary material available at 10.1038/s41598-024-76614-4.

## Introduction

The global COVID-19 pandemic has highlighted the importance of proactive surveillance and response to infectious diseases within communities. As a result, wastewater-based surveillance systems have gained significant attention in many countries, including the United States, Europe, and Japan, and are being actively incorporated into national infectious disease surveillance policies^1,2^.

Surveys for the detection of infectious pathogens in sewage are emerging as an innovative, multifaceted approach with broad implications for public health management. The World Health Organization (WHO), the U.S. National Institutes of Health (NIH) and various international organizations recognize the serious impact that infectious diseases have on communities around the world and are actively developing effective prevention and prediction systems^3^.

The sewage surveillance system is being studied based on reports that bacteria and viruses are present in sewage that local residents discharge through daily use. Pathogens can be detected using the nucleic acids present in sewage. Measuring the concentration of pathogens in sewage is an important indicator of the prevalence of pathogen infections in an area^4^.

In addition, the sewage monitoring system allows monitoring by period and region without infringing on individual privacy, enabling early detection of new infectious pathogens^5,6^.

In this study, we aimed to investigate and report the detection rates of 7 pneumonia-causing bacteria, 19 acute diarrhea-causing bacteria and viruses, 15 respiratory viruses, SARS-CoV-2, Zika virus, hepatitis A virus, poliovirus, Mpox, and measles among the most common infectious pathogens in Korea from December 2022 to November 2023 in sewage collected from six wastewater treatment plants (WWTPs) in Yongin city.

In particular, nucleic acid concentrations of 9 pathogens were additionally analyzed, including influenza A virus (IAV), human adenovirus (HAdV), human coronavirus (HCoV 229E, NL63, and OC43), human rhinovirus (HRV), SARS-CoV-2, *Campylobacter* spp., enteropathogenic *E. coli* (EPEC), norovirus GII (NoV GII), and sapovirus (SV). These pathogens were selected for comparison with the weekly pathogen and vector surveillance results released by the Korea Disease Control and Prevention Agency (KDCA). The respiratory virus data in the surveillance report and the pathogen data of acute diarrhea cases were derived from genetic testing results of samples collected from more than 70 medical institutions nationwide. Therefore, we would like to report by analyzing the correlation between the nucleic acid concentrations of the nine pathogens detected in sewage and the weekly pathogen and vector surveillance results announced by the KDCA.

## Results

PCR tests were performed for 47 species of pathogens during the study period, most of which pathogens associated with respiratory infections, diarrhea, and pneumonia were detected. However, Zika virus, hepatitis A virus (HAV), poliovirus, Mpox, and measles were never detected in sewage. Few clinical cases of Zika virus, HAV, poliovirus, Mpox, and measles were reported in the community during the study period. The absence of these pathogens in sewage aligns with the lack of clinical cases, indicating that there was no active transmission in the community at that time (Table 1).

**Table 1:** Types of viruses and bacteria analyzed in this study.

Types of viruses and bacteria	
Respiratory viruses	
Human adenovirus	Human bocavirus
Respiratory syncytial virus A	Human metapneumovirus
Respiratory syncytial virus B	Human coronavirus 229E
Influenza A virus	Human coronavirus NL63
Influenza B virus	Human coronavirus OC43
Human parainfluenza virus 1	Human rhinovirus A/B/C
Human parainfluenza virus 2	Human enterovirus
Human parainfluenza virus 3	SARS-CoV-2
Acute diarrhea-causing bacteria and viruses	
*Campylobacter* spp.	Enteropathogenic *E.coli* (eaeA)
*Clostridiuim difficile* toxin B	Enterotoxigenic *E.coli* (It/st)
*Salmonella* spp.	Enteroinvasive *E.coli* (aggR)
*Shigella* spp./EIEC	Group A rotavirus
*Vibrio* spp.	Norovirus GI
*Yersinia enterocolitica*	Norovirus GII
*Aeromonas* spp.	Astrovirus
*Clostridium difficile* hypervirulent	Enteric adenovirus
*Escherichia coli* O157	Sapovirus
Shiga toxin-producing *E*.*coli* (stx1/2)	
Pneumonia-causing bacteria	
*Mycoplasma pneumonia*	*Bordetella pertussis*
*Legionella pneumophila*	*Chlamydophila pneumonia*
*Streptococcus pneumoniae*	*Bordetella parapertussis*
*Haemophilus influenzae*	
Others	
Hepatitis A virus	Poliovirus
Monkeypox virus	Measles virus
Zika virus	

### Detection rate of respiratory viruses

The detection results are expressed as the ratio of the number of detections for each respiratory virus out of 24 total detections. This is the result of analyzing the influent water from six WWTPs for one year, twice a month, for a total of 24 times. Through this analysis, among 15 types of respiratory viruses, human bocavirus (HBoV) and SARS-CoV-2 were detected 24 times (100%) in six WWTPs. HAdV was most frequently detected in WWTP D (96%), followed by WWTP A (79%), WWTP E (79%), WWTP C (75%), WWTP B (58%), and WWTP F (58%).

Compared to those of other wastewater treatment plants, WWTP D exhibited the highest detection rate of HAdV. The next most frequently detected viruses were human enterovirus (HEV) (82%), HAdV (74%), IAV (63%), and HCoV OC43 (60%) for the pooled data of six WWTPs. Influenza B virus (IBV)(1%), respiratory syncytial virus A (RSV A) (3%), and human metapneumovirus (HMPV) (3%) were rarely detected during the study period. Human parainfluenza virus 1 (HPIV 1), human parainfluenza virus 2 (HPIV 2), and human parainfluenza virus 3 (HPIV 3) were detected irregularly in sewage samples, and HPIV 3 (37%) had a higher rate of detection than HPIV 1 (5%) and HPIV 2 (11%) (Table 2).

**Table 2:** Detection rates of respiratory virus in wastewater samples collected from six WWTPs in Yongin, Korea.

Viruses	PCR positive samples by WWTP location, *n* (%)						
	A (24)^*^	B (24)^*^	C (24)^*^	D (24)^*^	E (24)^*^	F (24)^*^	Pooled (144)^*^
Human adenovirus	19 (79)	14 (58)	18 (75)	23 (96)	19 (79)	14 (58)	107 (74)
Respiratory syncytial virus A	1 (4)	1 (4)	1 (4)	1 (4)	0 (0)	1 (4)	5 (3)
Respiratory syncytial virus B	2 (8)	2 (8)	4 (17)	3 (13)	1 (4)	4 (17)	16 (11)
Influenza A virus	15 (63)	14 (58)	18 (75)	17 (71)	14 (58)	13 (54)	91 (63)
Influenza B virus	0 (0)	0 (0)	1 (4)	0 (0)	0 (0)	0 (0)	1 (1)
Human parainfluenza virus 1	2 (8)	1 (4)	2 (8)	2 (8)	0 (0)	0 (0)	7 (5)
Human parainfluenza virus 2	5 (21)	3 (13)	2 (8)	1 (4)	1 (4)	4 (17)	16 (11)
Human parainfluenza virus 3	9 (38)	8 (33)	9 (38)	8 (33)	10 (42)	9 (38)	53 (37)
Human bocavirus	24 (100)	24 (100)	24 (100)	24 (100)	24 (100)	24 (100)	144 (100)
Human metapneumovirus	1 (4)	1 (4)	0 (0)	1 (4)	0 (0)	2 (8)	5 (3)
Human coronavirus 229E	13 (54)	11 (46)	12 (50)	13 (54)	10 (42)	13 (54)	72 (50)
Human coronavirus NL63	9 (38)	9 (38)	9 (38)	6 (25)	7 (29)	6 (25)	46 (32)
Human coronavirus OC43	16 (67)	17 (71)	14 (58)	13 (54)	11 (46)	15 (63)	86 (60)
Human rhinovirus A/B/C	13 (54)	12 (50)	12 (50)	11 (46)	13 (54)	16 (67)	77 (53)
Human enterovirus	20 (83)	20 (83)	22 (92)	21 (88)	15 (63)	20 (83)	118 (82)
SARS-CoV-2	24 (100)	24 (100)	24 (100)	24 (100)	24 (100)	24 (100)	144 (100)

Notes: ^*^No. of samples collected.

### Detection rate of acute diarrhea-causing bacteria and viruses

Among the 13 types of bacteria that cause acute diarrhea, *Aeromonas* spp., EPEC, and enterotoxigenic *E. coli* (ETEC) were consistently detected in all six WWTPs. The next most commonly found were enteroinvasive *E. coli* (EAEC) (99%), *Clostridium difficile* toxin B (C. *difficile* toxin B) (98%), and *Campylobacter* spp. (97%). *Shigella* spp./EIEC (1%) and *Clostridium difficile* hypervirulent (0%) were rarely identified. *Salmonella* spp. (44%), *Vibrio* spp. (33%), and *Yersinia enterocolitica* (54%) were intermittently detected in sewage samples. Among the six types of diarrhea viruses, NoV GII had the highest detection rate (98%). The detection rate of Group A rotavirus and astrovirus was 86%. Additionally, NoV GI (80%) and enteric adenovirus (78%) were found, while SV (35%) was irregularly detected (Table 3).

**Table 3:** Detection rates of acute diarrhea-causing bacteria and viruses in wastewater samples collected from six WWTPs in Yongin, Korea.

Bacteria and viruses	PCR positive samples by WWTP location, *n* (%)						
	A (24)^*^	B (24)^*^	C (24)^*^	D (24)^*^	E (24)^*^	F (24)^*^	Pooled (144)^*^
*Campylobacter* spp.	23 (96)	23 (96)	24 (100)	24 (100)	23 (96)	22 (92)	139 (97)
*Clostridium difficile* toxin B	22 (92)	24 (100)	24 (100)	24 (100)	24 (100)	23 (96)	141 (98)
*Salmonella* spp.	12 (50)	12 (50)	6 (25)	13 (54)	15 (63)	6 (25)	64 (44)
*Shigella* spp./EIEC	0 (0)	0 (0)	0 (0)	1 (4)	0 (0)	0 (0)	1 (1)
*Vibrio* spp.	5 (21)	7 (29)	10 (42)	10 (42)	5 (21)	11 (46)	48 (33)
*Yersinia enterocolitica*	12 (50)	13 (54)	15 (63)	13 (54)	14 (58)	11 (46)	78 (54)
*Aeromonas* spp.	24 (100)	24 (100)	24 (100)	24 (100)	24 (100)	24 (100)	144 (100)
*Clostridium difficile* hypervirulent	0 (0)	0 (0)	0 (0)	0 (0)	0 (0)	0 (0)	0 (0)
*Escherichia coli* O157	13 (54)	12 (50)	16 (67)	19 (79)	15 (63)	13 (54)	88 (61)
Shiga toxin-producing *E.coli*	18 (75)	13 (54)	19 (79)	20 (83)	15 (63)	17 (71)	102 (71)
Enteropathogenic *E.coli* (eaeA)	24 (100)	24 (100)	24 (100)	24 (100)	24 (100)	24 (100)	144 (100)
Enterotoxigenic *E.coli* (It/st)	24 (100)	24 (100)	24 (100)	24 (100)	24 (100)	24 (100)	144 (100)
Enteroinvasive *E.coli* (aggR)	23 (96)	24 (100)	24 (100)	24 (100)	24 (100)	24 (100)	143 (99)
Group A rotavirus	23 (96)	23 (96)	23 (96)	21 (88)	14 (58)	20 (83)	124 (86)
Norovirus GI	18 (75)	23 (96)	20 (83)	19 (79)	15 (63)	20 (83)	115 (80)
Norovirus GII	24 (100)	24 (100)	24 (100)	24 (100)	22 (92)	23 (96)	141 (98)
Astrovirus	21 (88)	20 (83)	21 (88)	22 (92)	20 (83)	20 (83)	124 (86)
Enteric adenovirus	23 (96)	17 (71)	22 (92)	22 (92)	18 (75)	10 (42)	112 (78)
Sapovirus	9 (38)	8 (33)	8 (33)	9 (38)	8 (33)	9 (38)	51 (35)

Notes: ^*^No. of samples collected.

### Detection rate of pneumonia-causing bacteria

Among the seven types of pneumonia-causing bacteria, *Haemophilus influenzae* was detected at nearly all six WWTPs and accounted for 99% of the cases. *Streptococcus pneumoniae* also showed a high detection rate, with 92% in WWTP A, 92% in WWTP B, 96% in WWTP C, 83% in WWTP D, 88% in WWTP E, and 92% in WWTP F. The pooled data for *Legionella pneumophila* indicated a detection rate of 23%, with intermittent occurrences. *Bordetella parapertussis* (8%), *Chlamydophila pneumoniae* (3%), *Mycoplasma pneumoniae* (1%), and *Bordetella pertussis* (1%) were rarely detected in all WWTPs during the study period (Table 4).

**Table 4:** Detection rates of acute pneumonia-causing bacteria in wastewater samples collected from six WWTPs in Yongin, Korea.

Bacteria	PCR positive samples by WWTP location, *n* (%)						
	A (24)^*^	B (24)^*^	C (24)^*^	D (24)^*^	E (24)^*^	F (24)^*^	Pooled (144)^*^
*Mycoplasma pneumonia*	0 (0)	0 (0)	2 (8)	0 (0)	0 (0)	0 (0)	2 (1)
*Legionella pneumophila*	6 (25)	4 (17)	4 (17)	8 (33)	5 (21)	6 (25)	33 (23)
*Streptococcus pneumoniae*	22 (92)	22 (92)	23 (96)	20 (83)	21 (88)	22 (92)	130 (90)
*Haemophilus influenzae*	24 (100)	24 (100)	24 (100)	24 (100)	24 (100)	23 (96)	143 (99)
*Bordetella pertussis*	0 (0)	1 (4)	0 (0)	0 (0)	0 (0)	1 (4)	2 (1)
*Chlamydophila pneumoniae*	3 (13)	0 (0)	0 (0)	0 (0)	0 (0)	1 (4)	4 (3)
*Bordetella parapertussis*	2 (8)	4 (17)	2 (8)	1 (4)	0 (0)	3 (13)	12 (8)

Notes: ^*^No. of samples collected.

### Comparison of trends between detection data in sewage by period and rate of pathogen detection in clinical samples

**Figure 1:**
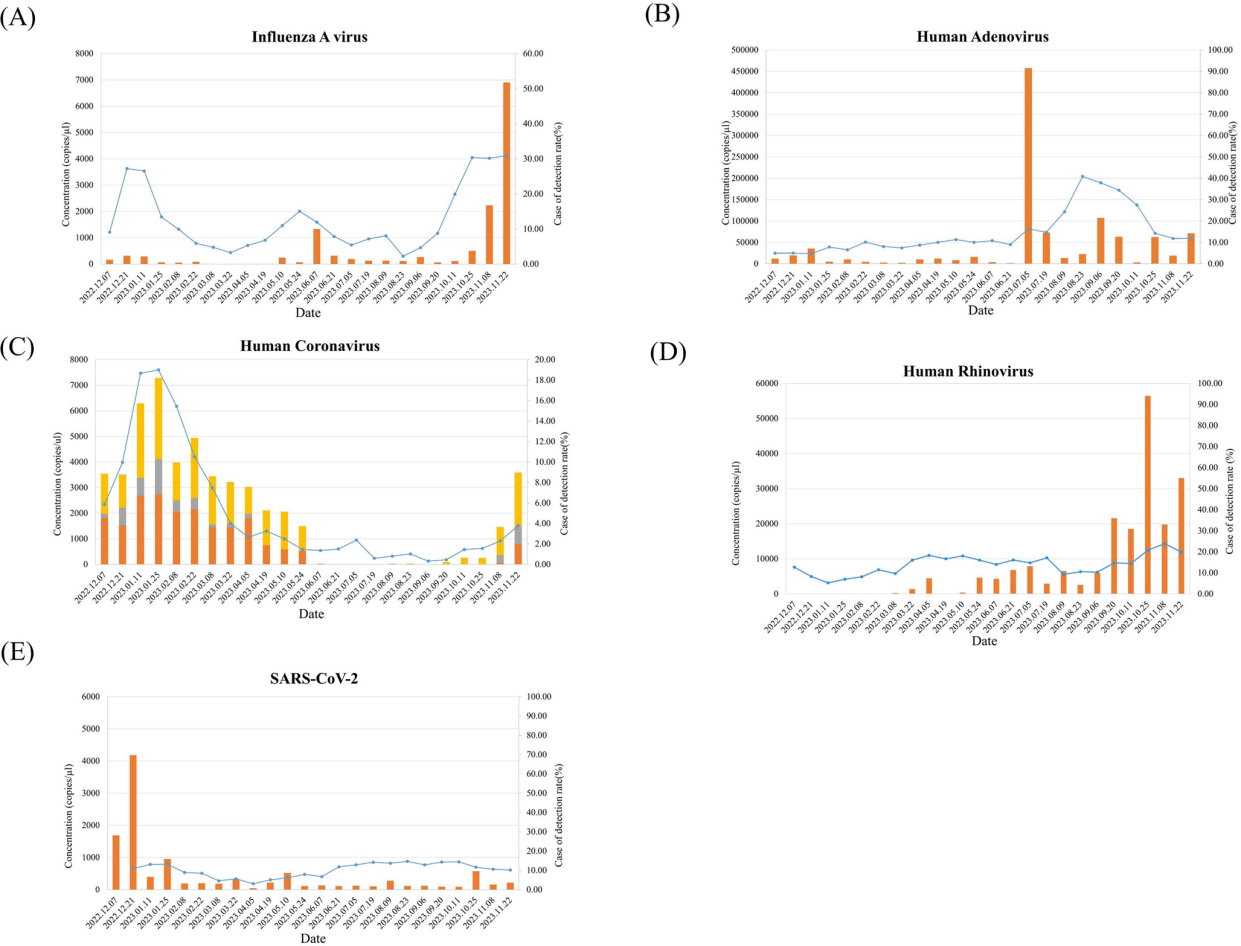
Correlation between the weekly rate of detection for respiratory viruses from the KDCA and the concentration of viruses detected at the six WWTPs. The rate of detection for each respiratory virus reported weekly by the KDCA and the concentration of each virus detected at the six WWTPs were analyzed and are presented in a graph. The orange bar represents the concentration (copies/µl) of the virus detected in sewage collected twice a month, and the blue line represents the rate of virus detection reported weekly by the KDCA. (**A**) Influenza A virus, (**B**) human adenovirus, (**C**) human coronavirus (229E, NL63, and OC43), (**D**) human rhinovirus A/B/C, and (**E**) SARS-CoV-2. For human coronavirus, three subtypes were analyzed (shown in orange for human coronavirus 229E, gray for OC43, and yellow for NL64), and the viral concentrations of human coronavirus represent the sum of the three types. The trend in the concentrations of four respiratory viruses, excluding SARS-CoV-2, was very similar to the trend in the rates of detection reported by the KDCA.

**Figure 2:**
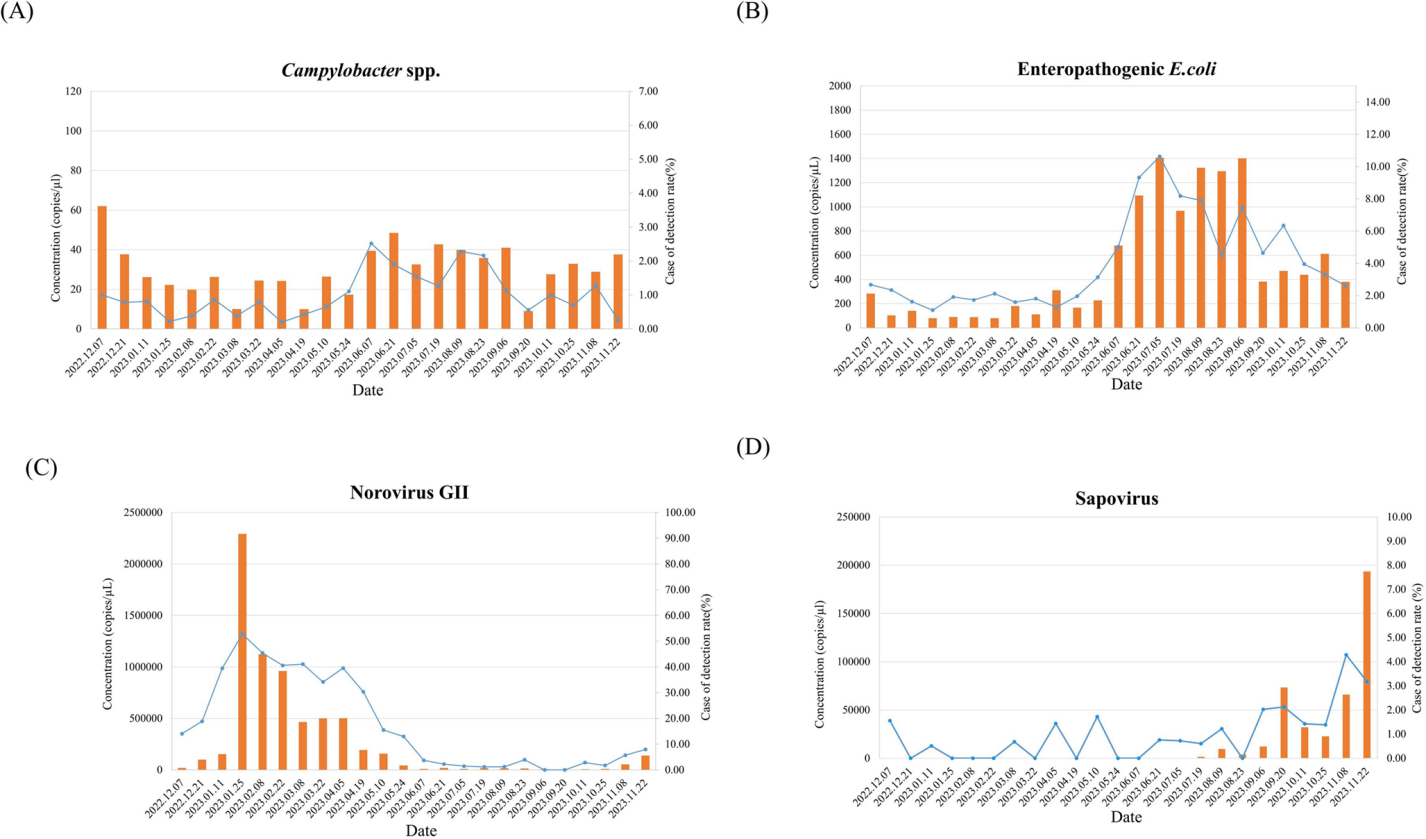
Correlation between the weekly rate of detection for pathogens causing acute diarrheal disease from KDCA and the concentration of pathogens detected in the six WWTPs. The weekly detection rate of each pathogen causing acute diarrheal disease by the KDCA report and the concentration of each pathogen detected in the six WWTPs were analyzed and are presented in a graph. The orange bar represents the concentration (copies/µL) of pathogens detected in sewage collected twice a month, and the blue line represents the rate of pathogen detection reported weekly by the KDCA. (**A**) *Campylobacter* spp., (**B**) enteropathogenic *E. coli* (eaeA), (**C**) norovirus GII, and (**D**) sapovirus. The trend in the concentrations of the four causative agents of acute diarrheal disease detected in sewage was very similar to the trend in the rates of detection reported by the KDCA.

Considering the change in nucleic acid concentration in sewage for each pathogen during the study period, the concentration of IAV was mainly high in sewage during the cold season, from November to December, and showed a sharp increase, especially toward the end of October. It was barely detected in March and April (Fig. 1a). In the case of HAdV, a respiratory virus, the concentration was low from February to June, but in the fall (September), the increase was greater than before (Fig. 1b). For HCoV, the analysis was conducted by summing the concentrations of three subtypes (229E, NL63, and OC43). They were rarely detected from June to September, and mainly in winter (November to February), when the concentration in sewage was significantly greater than in other seasons. Among the three subtypes, 229E and OC43 had higher concentrations than NL63, indicating that these two subtypes are dominant (Fig. 1c). The concentration of HRV in sewage was greater in fall and winter (September to November) than in spring and summer. The trend toward an increase or decrease in the nucleic acid concentration in sewage was similar to that reported by the KDCA (Fig. 1d). For diarrhea-causing bacteria and viruses, it was confirmed that when the detection rates of NoV GII, EPEC, and SV increased rapidly, the concentration in sewage also increased significantly (Fig. 2). Compared with other pathogens, the concentrations of *Campylobacter* spp. were generally similar throughout the study period, and the slight increasing/decreasing trend was similar to the weekly pathogen detection rate reported by the KDCA (Fig. 2).

**Figure 3:**
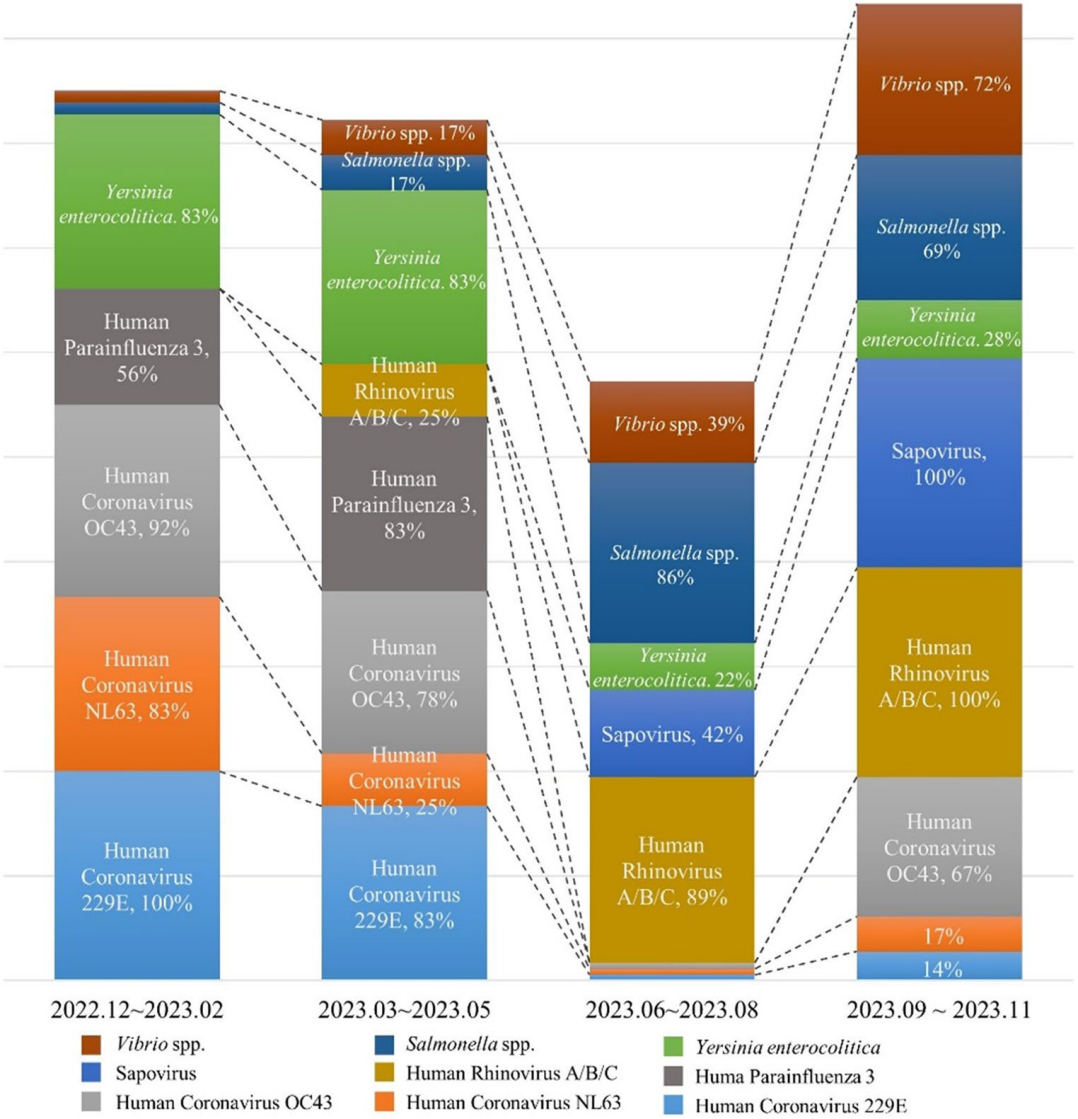
Trends in the rates of detection of pathogens in sewage by season. The graph shows the trend of the rates of detection of each pathogen in sewage by season. For each pathogen, the change in the rate of detection over 3 months is shown. In the case of human coronavirus (229E, NL63, and OC43), human parainfluenza virus 3, and *Yersinia enterocolitica*, the detection rates tended to decrease as summer approached, while for the detection rate of human rhinovirus A/B/C, *Salmonella* spp. and *Vibrio* spp. trended to gradually increase in summer.

Moreover, some microorganisms had higher or lower rates of detection for certain conditions (Fig. 3). HCoV (229E, NL63, OC43) showed high rates of detection in winter and early spring, demonstrated their rates of detection decreased sharply in summer their detection (to 3% for all three strains) and increased again in the fall. HPIV 3 showed a high rate of detection 83% in spring but had hardly detected in summer or fall. In contrast, HRV A/B/C showed a 25% rate of detection in spring but increased significantly to 89% and 100% in summer and fall, respectively. These changes may be related to the seasonal transmission pattern of the virus.

Among the pathogens causing acute diarrhea, *Y*. *enterocolitica* was detected at a high rate of 83% in spring and winter but decreased to 22% in summer and slightly increased to 28% in fall.

In contrast, few SV were detected in spring and winter, but the detection rate increased to 42% in summer and 100% in autumn. *Salmonella* spp. (86%) and *Vibrio* spp. (72%) were highly abundant in summer but decreased in winter. This is consistent with the fact that warmer temperatures generally promote the growth of these microorganisms.

### Correlation analysis between the nucleic acid concentration of pathogens detected in sewage and the pathogen rate of detection reported by the KDCA

Based on the detection results of viruses and bacteria during the study period, the correlation between the rate of detection for pathogens reported weekly by the KDCA and the nucleic acid concentration of sewage (copies/µl) was evaluated. The concentration of each pathogen was converted to copies from the Ct value using the standard curve. The concentrations obtained from the six WWTPs were pooled. The positivity rate of pathogens was obtained from the KDCA data. The correlation of these two values was evaluated using Spearman rank correlation. Analysis of the correlation between the nucleic acid concentration in sewage and the rate of detection reported by the KDCA revealed a correlation, except for that of SARS-CoV-2 (Fig. 1e). This study yielded very significant results between the nucleic acid concentration of pathogens detected in sewage and the pathogen detection rate reported by the KDCA.

IAV, HAdV, and HRV were moderately correlated, with rho values of 0.57, 0.45, and 0.58, respectively. For *Campylobacter* spp. and SV, the rho values were 0.62 and 0.63, respectively, indicating a strong correlation. When the concentrations of the three subtypes of HCoV 229E, NL63, and OC43 were combined and compared, the rho value was 0.90, indicating a very strong correlation. Additionally, EPEC and NoV GII were very strong correlated, with rho values of 0.86 and 0.92, respectively (Table 5). All the significant pathogens had p-values less than 0.05, confirming a positive correlation (Supplementary Table S3).

**Table 5:** Results of the statistical analysis between the concentration of pathogens in sewage and the results of the PVSWR provided by the KDCA.

Microorganisms type (*n*)	Correlation result (Spearman’s rho)		Grade
	ρ	p value	
Influenza A virus (24)	0·57	< 0·01	Moderate
Human adenovirus (24)	0·45	0·03	Moderate
Human coronavirus 229E/NL63/OC43 (24)	0·90	< 0·01	Very strong
Human rhinovirus (24)	0·58	< 0·01	Moderate
SARS-CoV-2 (24)	−0·18	0·4	Very weak
*Campylobacter* spp. (24)	0·62	< 0·01	Strong
Enteropathogenic *E.coli* (EPEC) (24)	0·86	< 0·01	Very strong
Norovirus GII (24)	0·92	< 0·01	Very strong
Sapovirus (24)	0·63	< 0·01	Strong

Abbreviations: ρ: Spearman correlation coefficient; p value: probability value.

Notes: A p-value less than 0.05 is typically considered evidence of a statistically significant correlation, meaning that it is unlikely that the observed correlation is due to random chance.

For these eight pathogens, all p-values were less than 0.05, satisfying the hypothesis of a positive correlation. However, for SARS-CoV-2, the rho value was − 0.18, and the p-value was 0.4, making it difficult to observe a correlation (Supplementary Table S3). It was confirmed that there was no strong association between the national data and Yongin city sewage data.

## Discussion

In this study, the presence of various pathogens, such as respiratory viruses, pneumonia-causing bacteria, acute diarrhea-causing bacteria and viruses, was confirmed in sewage collected from six WWTP sites in Yongin, Gyeonggi-do, Korea. Specifically, this study significantly expanded the sewage surveillance analysis for a total of 47 pathogens, including 15 respiratory viruses, 7 pneumonia-causing bacteria, 19 acute diarrhea-causing viruses and bacteria, SARS-CoV-2, Zika virus, HAV, poliovirus, Mpox, and measles, among other collected sewage.

In this study, HBoV and SARS-CoV-2 were consistently detected among 16 respiratory viruses, and HAdV, IAV, and HEV also exhibited high detection rates. In contrast, RSV A, IBV, and HMPV were infrequently detected. The WWTP D area showed a particularly high detection rate of HAdV. This is thought to have been influenced by the number of infants and young children^7^. In the case of the WWTP D region, there were more residential areas compared to other regions, and the number of populations was much higher than other regions. In particular, the population of infants and young children (0–14 years old) was 4 to 10 times higher than in other regions^8^. For this reason, it is hypothesized that HAdV, which mainly infects infants and young children, showed the highest detection rate in the WWTP D region.

In particular, the 100% rate of detection for HBoV was consistent with the findings of a recent community surveillance report in which 100% of HBoV, HRV, and parechovirus were detected in a study of 13 respiratory viruses in sewage samples for 6 months in Queensland, Australia^9^. Several studies have been reported the presence of various pathogens, including RSV, influenza, HPIV, and HRV, in wastewater, in addition to SARS-CoV-2^10–15^.

Among the bacteria that cause pneumonia, *H. influenzae* was consistently detected in nearly all the sewage samples, while *S. pneumoniae* showed varying rates of detection depending on the WWTP. Additionally, *L. pneumophila* was irregularly detected, and other pneumonia-causing bacteria were rarely observed. There are still limited data on sewage analysis for pneumonia-causing bacteria, so further research is necessary to improve the accuracy and reliability of the data obtained from sewage analysis.

In this study, in addition to respiratory viruses, bacteria and viruses that cause acute diarrhea were also analyzed. Among the 13 types of diarrhea-causing bacteria, *Aeromonas* spp., EPEC, and ETEC (IT/st) were detected consistently, while EAEC (aggR), *C*. *difficile* toxin B, and *Campylobacter* spp. also exhibited high detection rates. In contrast, *Shigella* spp./EIEC and *C. difficile* hypervirulent were rarely detected. These results are consistent with the report that the eae gene of EPEC was detected in all samples as a result of *E. coli* analysis (EPEC, EHEC) in sewage from Honolulu, Hawaii^16^.

Additionally, in this study, the diarrhea virus NoV GII had the highest detection rate among acute diarrhea-causing viruses, ranging from 92 to 100% in the six WWTPs. Enteric adenovirus, group A rotavirus, astrovirus, and NoV GI also showed high detection rates, but SV was irregularly detected in sewage samples. These results were consistent with reports in Sweden in which NoV GI was detected at 73% and NoV GII at 100% through virus analysis in sewage^17^. Moreover, the trend of increasing concentrations of NoV GII during the cold season was also observed^17^. The results of enteric adenovirus were similar to those of a study in which adenoviruses were detected in 88.7% of the sewage samples from Milan, Italy. The virus was found to be more closely related to diarrhea-causing pathogens than to respiratory pathogens, making it suitable for the surveillance of gastrointestinal infections^18^.

According to data reported by the Korea Centers for Disease Control and Prevention in 2022–2023, acute diarrhea-causing bacteria and viruses are seasonal, and norovirus is usually prevalent from winter to spring and bacteria such as pathogenic *E. coli* are prevalent in summer^19^. In addition, it is known that the prevalence of respiratory viruses also shows seasonality^20^. We think that the prevalence of these pathogens could be confirmed by sewage analysis, and the pathogens showing good seasonality were selected from the sewage data and plotted as a graph. In this sewage study, acute diarrhea-causing bacteria (EPEC, Vibrio, Salmonella, etc.) began to increase in spring and tended to spread from summer to autumn. In the case of norovirus, the cause of acute diarrhea, the concentration of virus in sewage increased mainly in winter rather than in summer. This was consistent with the results of the surveillance report in South Korea, which reported that bacterial-induced acute diarrheal disease frequently occurs in summer and autumn, and that there is a high prevalence of the virus in spring and winter^19^. In the case of para-influenza, it was mainly detected in sewage from winter to spring during the study period (2022–2023), which was inconsistent with the generally known prevalence of para-influenza, from April to August. However, in Korea from 2022 to 2023, there have been reports that para-influenza has rather increased detection rates in winter since the outbreak of COVID-19^20^. This finding was similar to the results of the present study. Therefore, monitoring various pathogens in sewage may be useful for monitoring pathogens that are prevalent mainly seasonally in Korea.

Notably, this study included an additional analysis of nucleic acid concentrations for 9 specific pathogens that can be compared with the weekly pathogen and vector surveillance results published by the KDCA. We analyzed the correlation between the nucleic acid concentrations of the 9 pathogens detected in sewage and the weekly pathogen and vector surveillance results announced by the KDCA. These pathogens include influenza A virus (IAV), human adenovirus (HAdV), human coronavirus (HCoV 229E, NL63, and OC43), human rhinovirus (HRV), SARS-CoV-2, *Campylobacter* spp., enteropathogenic *E. coli* (EPEC), norovirus GII (NoV GII), and sapovirus (SV). This study revealed significant correlations between the virus concentrations of eight pathogens, excluding SARS-CoV-2, detected in sewage collected from six WWTPs, and the weekly detection rates of these viruses reported by the KDCA. This highlights the usefulness of sewage surveillance tools and is consistent with other studies showing that sewage has potential utility in monitoring pathogens^21,22^.

However, in the case of SARS-CoV-2, the results did not show evidence of a correlation. The national clinical data on SARS-CoV-2 reported by the KDCA did not align well with some sewage data in the Yongin city area. Other studies have reported variations in the amount and duration of viral RNA shedding by infected individuals due to this cause. It has been reported that this variability may affect the concentration of viral RNA detected in wastewater samples and may not be directly correlated with the number of clinical cases^23,24^. In this study, it was not possible to confirm the correlation between the SARS-CoV-2 concentration (Ct value) detected in domestic sewage and the prevalence of COVID-19. However, monitoring of SARS-CoV-2 mutant strains in local domestic sewage through Nanopore Sequencing, another study currently underway within the institution, was conducted. It was verified that the analysis results and the variants at a specific time announced in the KDCA’s SARS-CoV-2 monitoring data were consistent (Supplementary information: Sequencing analysis).

In other words, the ‘FLip variation’, a specific mutation of HK.3 (a sublineage of EG.5), was confirmed in sewage collected from September 6, 2023, in Yongin city. Afterward, in November 2023, HK.3, a sublineage of EG.5, became dominant in the KDCA’s SARS-CoV-2 classification and monitoring data (Korea Disease Control and Prevention Agency COVID-19 Genetic Surveillance, https://www.kdca.go.kr). By comparing the SARS-CoV-2 mutant strains detected in sewage with the monthly SARS-CoV-2 mutation trend, it is possible to identify the timing of occurrence of specific mutations and predict mutations in major strains of the virus circulating in a specific region (Supplementary Figs. S2 and S3)^25^.

Unfortunately, this study is limited in that it was not able to directly compare the results with community clinical data from six WWTP areas where wastewater was collected. This was not easy to compare because there were no published clinical data from each community. A comparison of the results of sewage pathogen analysis in some areas (Yongin city) with the results of national infectious disease surveillance samples is clearly limited.

We also know that there is a limitation in not fully reflecting the difference in inflows according to the sewage treatment plant and not reflecting the various types of buildings distributed in each sewage treatment plant area. Therefore, in future studies, it is thought that more accurate data could be grasped only by analyzing sewage collected by building (e.g., schools, hospitals, shopping centers, etc.).

Despite these limitations, in the case of infectious disease surveillance using sewage, patient surveillance will be possible without infringing on individual privacy. In addition, monitoring by period and region is possible, and it is expected that the cost reduction effect will be greater than that of clinical surveillance for the purpose of prior monitoring^21,26^. Furthermore, it will be possible to monitor not only SARS-CoV-2 but also various pathogens related to public health, such as viral mutations, antibiotic-resistant bacteria, and factors associated with waterborne diseases, in sewage.

Research on sewage-based surveillance, also known as wastewater-based epidemiology (WBE), has been consistently conducted overseas. Pathogen and health indicator monitoring can be performed by analyzing various types of information in sewage samples. The United States National Wastewater Surveillance System (NWSS) was established to monitor the presence of SARS-CoV-2 in wastewater. The NWSS released a study that tracks trends in viral concentrations in wastewater and provides lead times of up to two weeks before COVID-19 clinical cases peak^27^. In Australia (CSIRO) and the Netherlands, research has been published to establish a quarantine system that enables the preemptive detection of asymptomatic carriers of infectious diseases such as COVID-19. This is achieved by analyzing information on biomarkers present in sewage and bowel movements discharged by local residents with confirmed infectious diseases^21^.

Sewage-based biomechanics is a technology that is already being introduced in countries with serious public health issues related to drug problems. It is used as a means to prevent health risks to local residents and to monitor criminal organizations by tracking and analyzing sewage discharged after use by local residents. This helps calculate the usage amount and discharge area for drugs such as cocaine, ecstasy, and marijuana^28,29^.

In particular, since the onset of the COVID-19 pandemic in December 2019, the utilization of wastewater-based surveillance technology has expanded globally. This technology is used to monitor and manage symptomatic and asymptomatic infections. It serves as an auxiliary means of clinical surveillance, which typically detects only symptomatic infections^30^.

Since early 2020, when it was reported that the genetic signal of SARS-CoV-2 in sewage from six WWTPs in the Netherlands and Australia increased concurrently with the number of confirmed COVID-19 cases, sewage-based surveillance systems have garnered global attention^21,31^.

Since 2021, the KDCA has been conducting sewage-based infectious disease monitoring through a pilot project, and based on this, it is working with 18 public health and environmental research institutes across the country to carry out sewage-based infectious disease monitoring (KOWAS) projects including pathogens such as SARS-CoV-2, norovirus, human influenza virus (A and B), and antibiotic-resistant strains^32,33^. The system we have developed is easy and simple to detect pathogens in sewage, and since it has a system that can detect 47 different types of pathogens, we believe it will be of great help in expanding the sewage surveillance system of national organizations in the future.

Sewage generated from the community contains valuable information about the living patterns and health status of residents. Although the comparative analysis data were limited, the pathogens analyzed in this study were found to be present in the community or strongly suspected. Numerous studies have been conducted on surveillance systems using wastewater. However, there has never been a study in the world in which various pathogens (47 types) such as respiratory viruses, pneumonia-causing bacteria, diarrhea-causing bacteria, and viruses have been detected, as in this study. The research revealed that not only respiratory viruses but also pneumonia-causing bacteria, acute diarrheal pathogens, and viruses were detected in sewage. Moreover, a correlation was observed between the nucleic acid concentration and the incidence of infectious diseases.

This is the first time that such a wide-ranging infectious disease surveillance study has been attempted in Korea. The results of this study suggest the possibility of expanding the wastewater pathogen surveillance system in Korea, including wastewater collection, nucleic acid extraction, pathogen detection, and quantitative analysis. Additionally, to overcome the limitations of this study, it is anticipated that actively investigating the clinical data in community medical institutions in the future will enable a more accurate comparative analysis. This, in turn, will help in building a more effective wastewater monitoring system, aiding in predicting the prevalence of infectious diseases in the community.

## Methods

### Study design

This study was conducted by examining sewage from a WWTP in Yongin city to determine whether pathogens such as SARS-CoV-2, influenza, and acute diarrhea-causing pathogens were occurring and trending and whether they were prevalent. Through a sewage-based ‘Yongin Citizen Health Monitoring’ study, we identified the occurrence of infectious pathogens by testing for viruses and bacteria in domestic sewage. The analyzed pathogens included 15 types of respiratory viruses, 7 types of pneumonia-causing bacteria, 19 types of acute diarrhea-causing bacteria and viruses, SARS-CoV-2, Zika virus, hepatitis A virus (HAV), poliovirus, Mpox, and measles (Table 5). Nucleic acids from viruses or bacteria were extracted, and various pathogens were detected through real-time and conventional PCR. Additionally, for some pathogens, the concentration of the pathogen in sewage was calculated, and the correlation with the rate of detection for each pathogen reported by the KDCA was confirmed through statistical analysis.

### Sample collection and nucleic acid extraction

For one year, from December 2022 to November 2023, we collected site 24-hour composite influent wastewater from six WWTPs in Yongin city (A, B, C, D, E, and F; Supplementary Fig. S1) twice a month (a total of 24 times) at volume of 1.5 L. A total of 144 samples were analyzed.

For samples collected from a specific WWTP, sewage was concentrated, and nucleic acids were extracted using the MagMax Wastewater Ultra Nucleic Acid Isolation Kit with Virus Enrichment (Thermo Fisher Scientific™, Austin, USA) with a KingFisher Flex system (Thermo Fisher Scientific™, Marsiling, Singapore). In this kit, the sample is concentrated using magnetic beads, followed by nucleic acid extraction also utilizing magnetic beads. The experiment was conducted following the guidelines outlined in the user manual provided by the manufacturer (Supplementary information : Sample concentration and nucleic acid extraction).

### PCR analysis

In this study, a variety of pathogens were analyzed. The analysis method involved real-time PCR using a TaqMan probe and a Bio-Rad CFX96 thermal cycler (Bio-Rad Laboratories, Hercules, California, USA). Respiratory viruses, pneumonia-causing bacteria, acute diarrhea-causing bacteria and viruses, and the Zika virus were analyzed using a commercial assay kit following the manufacturer’s instructions (Supplementary information : PCR analysis).

For in-house developed assays, we utilized probe-based RT-PCR assays for HAV, measles virus, Mpox, and SARS-CoV-2 analysis. The base sequence was verified at the National Center for Biotechnology Information (NCBI), and primers and probes were designed using Primer3 and subsequently verified via in silico analysis utilizing BLAST tool which check their specificity and efficiency (Supplementary Table S1). The analysis of poliovirus was conducted using the conventional PCR method with a C1000 Thermal Cycler (Bio-Rad Laboratories).

Positive and negative controls were included throughout the entire process to ensure the reliability of the PCR and real-time PCR results. Additionally, PCR included internal controls targeting human genomic DNA to confirm the integrity of the process from nucleic acid extraction to PCR. The negative controls showed no amplification for all targets analyzed. Positive controls yielded the expected positive results, with consistent values measured for each PCR run.

Standard curves were established for IAV, HAdV, HCoV (229E, NL63, OC43), HRV, SARS-CoV-2, *Campylobacter* spp., EPEC, NoV GII, and SV, for which the data are available in the KDCA. We generated a standard curve using standard materials (whole genome) for bacteria and viruses (Supplementary Table S2). For SARS-CoV-2, we utilized RNA containing the target gene for PCR. A series of standards (10^0^ to 10^8^ copies/µl of standard materials) underwent PCR, and a standard curve was created based on the Ct values obtained. The slope of the standard curve ranged from − 3.21 to -3.66, confirming an amplification efficiency of more than 90%, and the correlation coefficient (r^2^) was also 0.97 or greater. Additional information on standard curves is provided in the supplementary information (Supplementary Table S4). This standard curve was used to determine the concentration of specific pathogens in sewage and compared the trends in pathogen concentration and positivity rates. The wastewater data (Ct values) obtained from real-time PCR were subsequently converted into concentrations (copies/µl).

### Statistical analysis

Spearman’s rank correlation (ρ) was utilized to examine the null hypothesis that the pathogen detection rate in clinical samples was not correlated with the concentration of the pathogen in domestic sewage. Due to the non-normal distribution of the variables, Spearman’s rank correlation was chosen. The pathogen detection rate in clinical samples was compared and analyzed with the weekly pathogen detection data provided by the KDCA. In the case of pathogen concentration values detected in domestic sewage, the pathogen concentrations in the sewage for each WWTP were first calculated and subsequently summed.

This correlation was confirmed by comparing the pathogen concentration data obtained from sewage samples with weekly detection rate of each pathogen reported by the KDCA. In the case of correlation analysis, all data from the 1st to the 24th were analyzed, and in the case of non-detect, whether it is clinical data or sewage data, it is marked as 0 and included in the analysis. Statistical analysis was performed using RStudio (R version 4.3.1), and the null hypothesis was rejected if the p-value was less than 0.05. Spearman’s rank correlation coefficient, rho, has a value between − 1.0 and + 1.0. The closer the rho value is to either + 1.0 or -1.0, the stronger the linear correlation between the two variables (Supplementary Table S3).

### Clinical data sources

Nine pathogens (IAV, HAdV, HCoV (229E, NL63, OC43), HRV, SARS-CoV-2, *Campylobacter* species, EPEC, NoV GII, SV) were selected out of 47 pathogens studied for comparison with the weekly pathogen and vector surveillance results published by KDCA. The Pathogen and Vector Surveillance Weekly Report (PVSWR), published weekly by the KDCA, was utilized the clinical data used in this study^34^. The respiratory virus data in the weekly surveillance reports were derived from genetic tests on suspected respiratory virus infection patients from 77 medical institutions nationwide conducted by the Infectious Disease Diagnostic Analysis Division of the KDCA^20,34^. In addition, data published by the KDCA using the number of reports of acute diarrhea disease from 70 medical institutions nationwide were utilized^19,34^. We analyzed the correlation by comparing disease surveillance data from KDCA with trends in pathogen concentrations detected in sewage during the study period.

## Supplementary Information

Supplementary Material 1.

## Data Availability

The main data supporting the results of this study are available within the paper and its Supplementary Information. The datasets used and/or analyzed during the current study available from the corresponding author on reasonable request. Pathogens & Vectors Surveillance Weekly Report for South Korea, are available from the KDCA at https://dportal.kdca.go.kr/pot/bbs/BD_selectBbsList.do?%20q_bbsSn=1010&q_bbsDocNo=&q_clsfNo=3&q_searchKeyTy=&q_searchVal=&q_currPage=1&q_sortName=&q_sortOrder=^34^. Sequence data that support the findings of this study have been deposited in the Genbank, NCBI with The Accession numbers [PQ151713-PQ151754]. https://www.ncbi.nlm.nih.gov/search/all/?term= The Accession numbers (Genbank, NCBI) of sequence data are attached in Supplementary Information Table 5.
