# Supplementary material for: Development of a wastewater based infectious disease surveillance research system in South Korea
Source: Sci Rep. 2024 Oct 19;14:24544. doi: 10.1038/s41598-024-76614-4 (PMC11490628; doi:10.1038/s41598-024-76614-4)
Supplement: Supplementary file 1 — Supplementary Material 1. [file 41598_2024_76614_MOESM1_ESM.docx]

**Supplementary information**

Development of a Wastewater Based Infectious Disease Surveillance Research System in South Korea

Yun-Tae Kim^1*^, Kyungwon Lee^2,3^, Hyukmin Lee^2^, Bokyung Son^1^, Myeongwon Song^1^, Seung-Hyun Lee^1^, Miran Kwon^1^, Dong-Soo Kim^4^, Tae-Hun Noh^4^, Sanghoo Lee^4^, Young-Jin Kim^3^, Mi-Kyeong Lee^3^, Kyoung-Ryul Lee^3^

1. Department of R＆D Innovation Center, Seoul Clinical Laboratories, Gyeonggi-do, Republic of Korea
2. Department of Laboratory Medicine, Research Institute of Bacterial Resistance, Yonsei University College of Medicine, Seoul, Republic of Korea
3. Seoul Clinical Laboratories, Gyeonggi-do, Republic of Korea
4. SCL Healthcare Inc. Gyeonggi-do, Republic of Korea

*** Corresponding author**: [ytkim@scllab.co.kr](mailto:ytkim@scllab.co.kr)

Number of pages: 8

Number of figures: 3

Number of tables: 3

**Sample concentration and nucleic acid extraction**

Sixty milliliters of each of the six sewage samples were placed into a 24 deep-well tip comb plate that was then mounted onto the KingFisher Flex system. Using enrichment beads, the sewage was concentrated to a final volume of 3 ml. Three milliliters of concentrated sewage was placed into a 24-deep-well tip comb plate that was then mounted onto the KingFisher Flex system. The nucleic acids were extracted using lysis buffer, proteinase K, binding solution, magnetic binding beads, wash buffer, and 80% ethanol. A final volume of 300 μl of nucleic acid was obtained through this process.

**PCR analysis**

Fifteen representative viruses of respiratory infections were analyzed by RT-qPCR using Allplex™ Respiratory Panel (Seegene Inc., Korea). The viruses analyzed included human adenovirus, influenza A virus, influenza B virus, respiratory syncytial virus A, respiratory syncytial virus B, human parainfluenza virus 1, human parainfluenza virus 2, human parainfluenza virus 3, human metapneumovirus, human bocavirus, human rhinovirus A/B/C, human coronavirus 229E, human coronavirus NL63, human coronavirus OC43, and human enterovirus.

Thirteen types of acute diarrhea-causing bacteria were analyzed by real-time PCR using the Allplex™ GI-Bacteria Assay Kit (Seegene Inc., Seoul, Korea).

The bacteria analyzed were *Campylobacter* spp., *Clostridium difficile* toxin B, *Salmonella* spp., *Shigella* spp./EIEC, *Vibrio* spp., *Yersinia enterocolitica, Aeromonas* spp*.,* EPEC (eaeA), enterotoxigenic *E. coli (ETEC)* (It/st), enteroaggregative *E. coli* (EAEC) (aggR*), Clostridium difficile* hyperviruent*, E. coli* O157, and Shiga toxin-producing *E. coli* (STEC) (stx1/2).

Six types of viruses causing acute diarrhea were analyzed by real-time PCR using the Allplex™ GI-Virus Assay kit (Seegene Inc., Korea). The viruses analyzed were rotavirus, norovirus GI, norovirus GII, astrovirus, enteric adenovirus, and sapovirus.

Seven types of pneumonia-causing bacteria were analyzed by real-time PCR using Allplex™

PneumoBacter Assay Kit (Seegene Inc., Korea). The bacteria analyzed were *Mycoplasma pneumoniae*, *Legionella pneumophila*, *Streptococcus pneumoniae*, *Haemophilus influenzae*, *Bordetella pertussis*, *Chlamydophila pneumoniae*, and *Bordetella parapertussis*.

Zika virus was analyzed by real-time PCR using a RealStar Zika Virus RT‒PCR Kit (Altona Diagnostics, Germany).

**Sequencing analysis**

In our study, domestic sewage collected every 2 weeks from 6 sewage treatment plants in urban region from June 2023 to November 2023 was used to concentrate and isolate viruses using MagMax wastewater ultra nucleic acid isolation kits with virus enrichment kits. Positive samples confirmed were subjected to SARS-CoV-2 whole genome sequencing (WGS) using Oxford Nanopore Technologies GridION. The experimental data was aligned through Epi2me, analyzed using Nextclade, that was compared with KDCA official reports and monthly COVID-19 monitoring results (total 780 cases) ongoing at SCL.

Nanopore sequencing is a representative of NGS long-read sequencing method that involves sequencing changes in electrical signals that appear when nucleic acids pass through proteins with nano-sized pores. In the case of SARS-CoV-2, the whole genome (30kb) was analyzed using nanopore sequencing. In this experiment, SQK-RBK110.96, EXP-MRT001, a commercialized kit of Oxford Nanopore Technologies, and the flow cell R.9.4.1 version were used.

**Tables S1. Primer & Probe sequence**

|  | Oligo | Sequence 5’-3’ | Fluorescence | Concentration | Run protocol |
| --- | --- | --- | --- | --- | --- |
| Measles Virus | N_F | TGGCATCTGAACTCGGTATCAC |  | 300nM | 1 step: 50℃ 10 min,  2 step: 95℃ 15 min  3 step: 40 cycles of  95℃ 5 sec and  60℃ 30 sec |
|  | N_R | TGTCCTCAGTAGTATGCATTGCAA |  |  |  |
|  | N_probe | CCGAGGATGCAAGGCTTGTTTCAGA | FAM | 250nM |  |
|  | H_F | GAGATCCATAAAAGCCTCAGYACC |  | 300nM |  |
|  | H_R | GCCCACTTCATCMCCGATG |  |  |  |
|  | H_probe | CTAACTCAATCGAGCATCAGGTCAAGGA | HEX | 250nM |  |
| Hepatitis A virus | HAV_F | CTCTTTGATCTTCCACAAGRGGT |  | 500nM | 1 step: 55℃ 1 hour  2 step: 95℃ 5 min  3 step: 45 cycles of  95℃ 15 sec and  60℃ 1 min and 65℃  1 min |
|  | HAV_R | GCCGCTGTTACCCTATCCAA |  | 900nM |  |
|  | HAV  _probe | AGGCTACGGGTGAAACCTCTTAGG | FAM | 150nM |  |
| Mpox | G2R-F | GGAACATATTCTCACACCGTCTC |  | 200nM | 1 step: 95℃ 10 min  2 step: 45 cycles of  95℃ 5 sec and  60℃ 20 sec and  72℃ 30 sec |
|  | G2R-R | GATACAGGTTAATTTCCACATCG |  |  |  |
|  | G2R-probe | CACAGATAAATGCGAACCCGTCGT | FAM | 100nM |  |
|  | F3L-F | CATCTATTATAGCATCAGCATCAGA |  | 200nM |  |
|  | F3L-R | GATACTCCTCCTCGTTGGTCTAC' |  |  |  |
|  | F3L-probe | TGTAGGCCGTGTATCAGCATCCATT' | FAM | 100nM |  |
| SARS-CoV-2 | orf1ab_F | CACCTAATAATACAGATTTTTCCAG |  | 300nM | 1 step: 25℃ 1 min,  2 step: 53℃ 8 min  3 step: 95℃ 1 min  4 step: 40 cycles of  95℃ 2 sec and  58℃ 20 sec |
|  | orf1ab_R | GAAGTCCTTTGTACATAAGTGGTA |  |  |  |
|  | orf1ab _Probe | AGTGCTAAACCACCGCCTGGAGA | FAM | 100nM |  |
| Internal control | H-Bg_F | TCCACTCCTGATGCTGTTATG |  | 200nM |  |
|  | H-Bg_R | CTTGAGGTTGTCCAGGTGAG |  |  |  |
|  | H-Bg  _probe | TCATGGCAAGAAAGTGCTCGGTGCCT | Texas Red | 100nM |  |
| Poliovirus | PV_F | GCGTGTAATGACTTCAGCGT |  | 450nM | 1 step: 60℃ 10 min,  2 step: 98℃ 2 min  3 step: 40 cycles of  95℃ 5 sec and  60℃ 30 sec  4 step: 72℃ 5 min |
|  | PV_R | TTGTAATCCACTCCAGGGCC |  |  |  |

* The limit of detection (LoD) was 10 copies/ul for all 5 in-house development PCR methods.

**Table S2. The information of Standard materials**

| Manufacturer Cat No. | Pathogen |
| --- | --- |
| KBPV-VR-32D | RNA from influenza A virus (H3N2) A/Korea/2007/H3N2(TC-adapted) |
| KBPV-VR-60D | DNA from adenovirus type 4 |
| KBPV-VR-8D | RNA from human coronavirus OC43 |
| KBPV-VR-9D | RNA from human coronavirus 229E |
| Twist, REF: 103027 | [Synthetic control] coronavirus NL63 |
| KBPV-VR-81D | RNA from human rhinovirus 1 |
| ATCC 700819D-5 | Genomic DNA from *Campylobacter jejuni* strain NCTC 11168 |
| ATCC 43888DX | *Escherichia coli*; O157 |
| ATCC VR-3235SD | Quantitative Synthetic RNA from norovirus G2 (II) |
| ATCC VR-3237SD | Quantitative Synthetic RNA from sapovirus |

**Table S3. Describing Correlation Coefficients**

| **Correlation Result (Spearman's rho)** | **Grade description** |
| --- | --- |
| +1.0 | Perfect positive association |
| +0.8 to 1.0 | Very strong + association |
| +0.6 to 0.8 | Strong + association |
| +0.4 to 0.6 | Moderate + association |
| +0.2 to 0.4 | Weak + association |
| 0.0 to +0.2 | Very weak + or no association |
| 0.0 to -0.2 | Very weak - or no association |
| -0.2 to – 0.4 | Weak - association |
| -0.4 to -0.6 | Moderate - association |
| -0.6 to -0.8 | Strong - association |
| -0.8 to -1.0 | Very strong - association |
| -1.0 | Perfect negative association |

The table above provides some guidelines on how to explain the strength of the correlation coefficient. These are just guidelines for explanation.

**Table S4. qPCR performance characteristics**

|  | Correlation coefﬁcient  (r^2^) | Slope | Y-intercept |
| --- | --- | --- | --- |
| Influenza A virus | 0.9945 | -3.5219 | 44.187 |
| Human adenovirus | 0.9934 | -3.2273 | 45.289 |
| Human coronavirus | 0.9734 | -3.655 | 45.93 |
| SARS-CoV-2 | 0.9985 | -3.2162 | 35.968 |
| Human rhinovirus | 0.9974 | -3.622 | 48.689 |
| *Campylobacter* spp. | 0.9824 | -3.5083 | 38.605 |
| Enteropathogenic *E.coli* | 0.996 | -3.5471 | 36.462 |
| Norovirus GII | 0.9819 | -3.5164 | 43.74 |
| Sapovirus | 0.9944 | -3.4141 | 44.735 |

**Table S5. GenBank accession number(s) for SARS-CoV-2 variants obtained from wastewater samples**

| **Accession number** | **Variant name** |
| --- | --- |
| PQ151713 | Korea:Giheung-WWTP:23.06.07:XBB.1.9.1 |
| PQ151714 | Korea:Gugal-WWTP:23.06.07:XBB |
| PQ151715 | Korea:Sanghyeon-WWTP:23.06.07:XBB.1.5 |
| PQ151716 | Korea:Yeongdeok-WWTP:23.06.07:XBB.1.22 |
| PQ151717 | Korea:Giheung-WWTP:23.06.21:XBB.1 |
| PQ151718 | Korea:Gugal-WWTP:23.06.21:XBB.1.16 |
| PQ151719 | Korea:Yongin-WWTP:23.06.21:XBB.1.5 |
| PQ151720 | Korea:Yeongdeok-WWTP:23.06.21:EG.2 |
| PQ151721 | Korea:Gugal-WWTP:23.07.05:XBB.1.9.1 |
| PQ151722 | Korea:Yongin-WWTP:23.07.05:XBB.1.9.1 |
| PQ151723 | Korea:Yongin-WWTP:23.07.19:XBB.1.9.1 |
| PQ151724 | Korea:Suji-WWTP:23.07.19:EG.2 |
| PQ151725 | Korea:Yeongdeok-WWTP:23.07.19:XBB.1.9.1 |
| PQ151726 | Korea:Gugal-WWTP:23.08.09:XBB.2.4 |
| PQ151727 | Korea:Yongin-WWTP:23.08.09:XBB.1.9.1 |
| PQ151728 | Korea:Sanghyeon-WWTP:23.08.09:XBB.1.5 |
| PQ151729 | Korea:Yeongdeok-WWTP:23.08.09:EG.2 |
| PQ151730 | Korea:Giheung-WWTP:23.08.23:XBB.1.22 |
| PQ151731 | Korea:Gugal-WWTP:23.08.23:XBB.1.9.1 |
| PQ151732 | Korea:Yongin-WWTP:23.08.23:EG.5.1 |
| PQ151733 | Korea:Suji-WWTP:23.08.23:XBB.2.4 |
| PQ151734 | Korea:Sanghyeon-WWTP:23.08.23:HF.1 |
| PQ151735 | Korea:Yeongdeok-WWTP:23.08.23:XBB.2.3.11 |
| PQ151736 | Korea:Giheung-WWTP:23.09.06:XBB.1.9.1 |
| PQ151737 | Korea:Gugal-WWTP:23.09.06:EG.5.1 |
| PQ151738 | Korea:Yongin-WWTP:23.09.06:XBB.1.22 |
| PQ151739 | Korea:Suji-WWTP:23.09.06:XBB.1.9.1 |
| PQ151740 | Korea:Sanghyeon-WWTP:23.09.06:FL.10 |
| PQ151741 | Korea:Yeongdeok-WWTP:23.09.06:EG.5.1.19 |
| PQ151742 | Korea:Gugal-WWTP:23.09.20:EG.5.1 |
| PQ151743 | Korea:Yongin-WWTP:23.09.20:XBB.1.9.2 |
| PQ151744 | Korea:Suji-WWTP:23.09.20:XBB.1.16 |
| PQ151745 | Korea:Giheung-WWTP:23.10.11:EG.5.1.1 |
| PQ151746 | Korea:Giheung-WWTP:23.10.25:EG.5.1.1 |
| PQ151747 | Korea:Gugal-WWTP:23.10.25:HK.3 |
| PQ151748 | Korea:Suji-WWTP:23.10.25:EG.5.1.1 |
| PQ151749 | Korea:Giheung-WWTP:23.11.08:HK.3 |
| PQ151750 | Korea:Gugal-WWTP:23.11.08:EG.5.1.1 |
| PQ151751 | Korea:Yongin-WWTP:23.11.08:EG.5.1.1 |
| PQ151752 | Korea:Yeongdeok-WWTP:23.11.08:HK.3 |
| PQ151753 | Korea:Gugal-WWTP:23.11.21:JF.1 |
| PQ151754 | Korea:Yongin-WWTP:23.11.21:HK.3 |

**
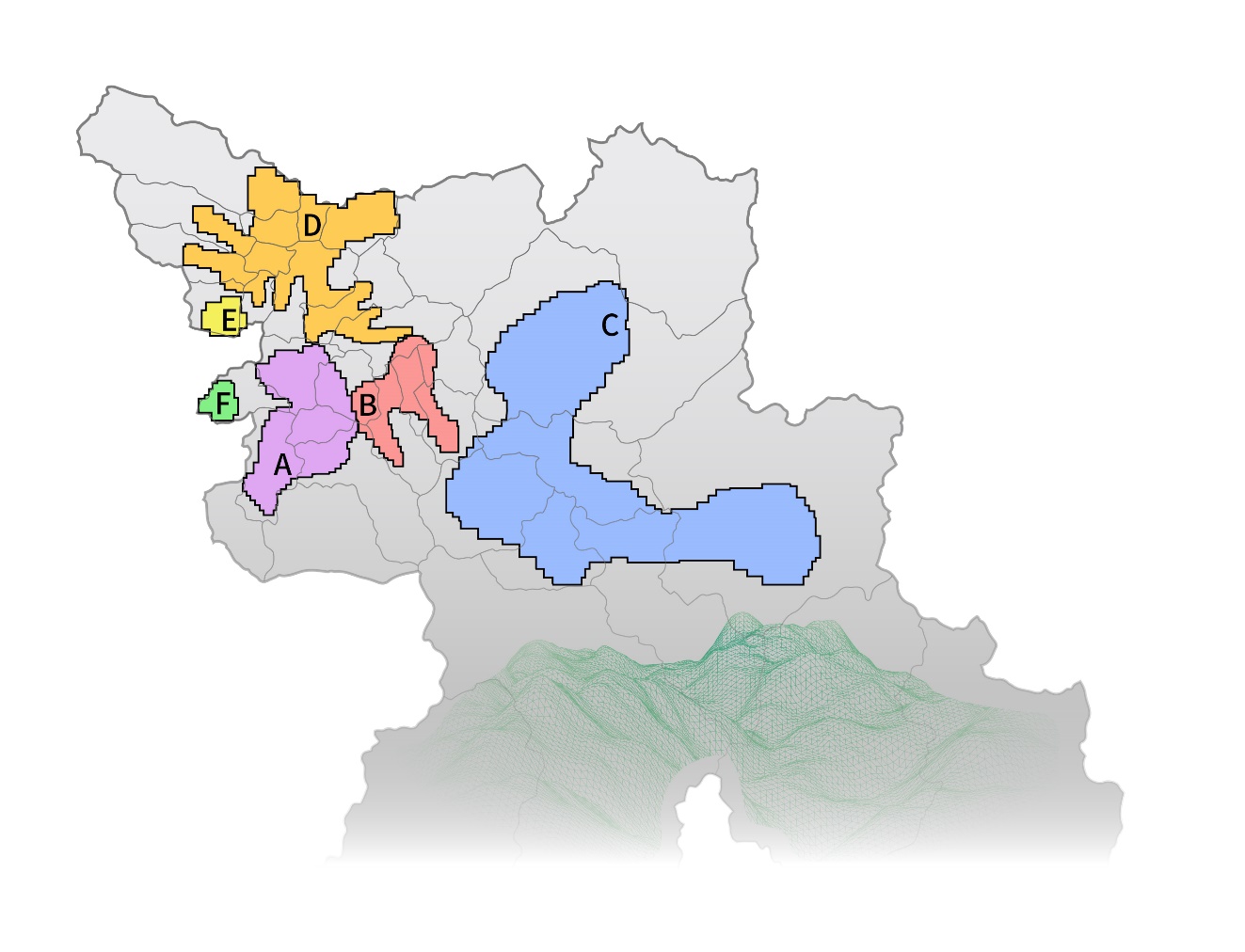
**

**Figure S1.** Location of the 6 wastewater treatment plants (WWTPs) included in the study**.**

WWTP A (residential & commercial area); Sewage treatment volume of 50,000 tons/day; 80,000 people
WWTP B (areas with a high density of hospitals); Sewage treatment volume 40,000 tons/day; 65,000 people
WWTP C (areas with a high density of schools); Sewage treatment volume 56,000 tons/day; 90,000 people
WWTP D (residential area with high housing density); Sewage treatment volume 150,000 tons/day; 310,000 people
WWTP E (residential & commercial area); Sewage treatment volume 13,000 tons/day; 30,000 people
WWTP F (residential & commercial area); Sewage treatment volume 13,000 tons /day; 30,000 people

<https://www.data.go.kr/data/3073222/fileData.do>

**
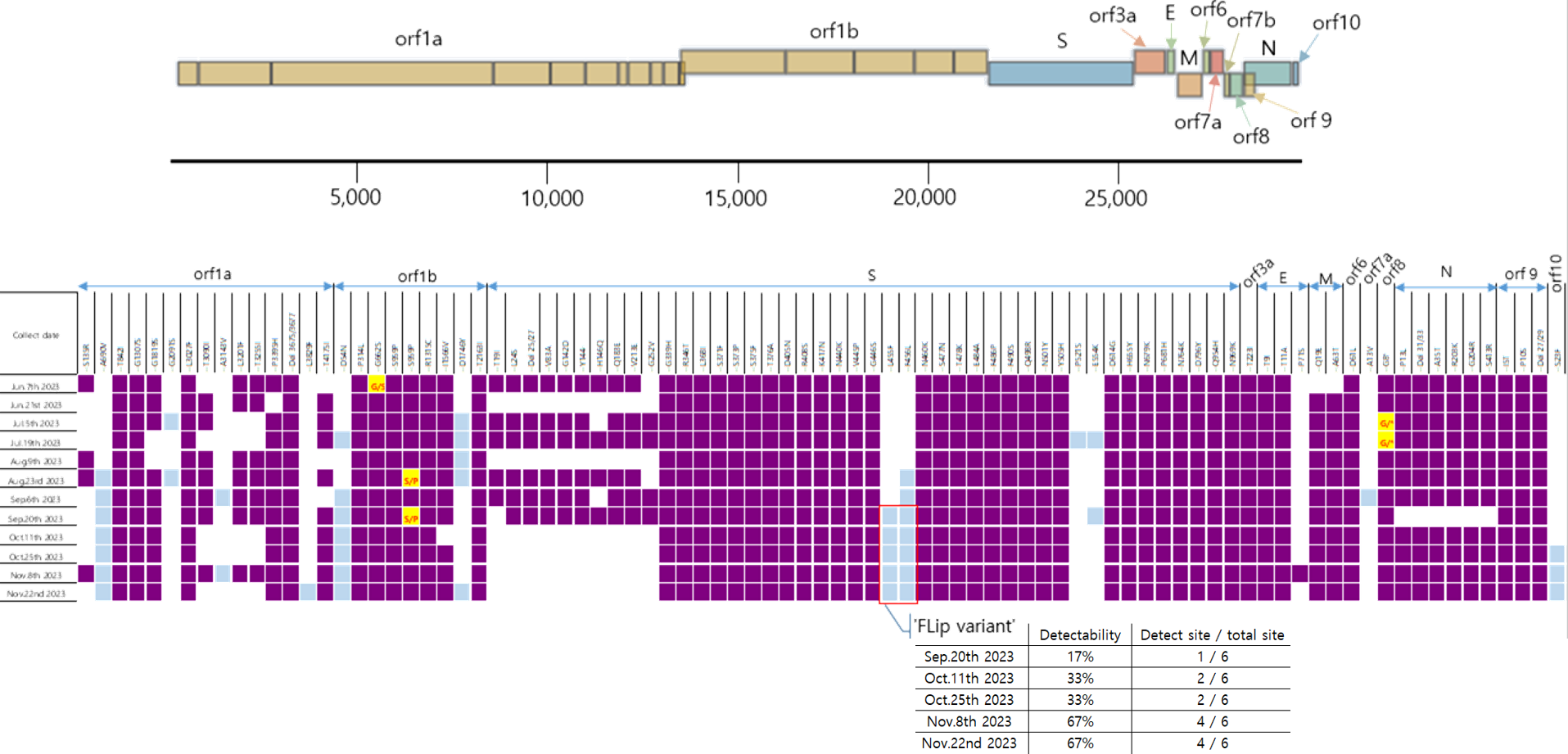
**

**Figure S2. Amino acid mutations in wastewater according to collection period.**

Various mutations were identified at each collection time and site, occurring new subvariants over time. The mutations (orf1a:A690V, S:F456L) sequentially confirmed from the sample collected on Aug. 23rd are specific mutations of XBB.1.9.2 and EG.5. In addition, the mutations orf1b: D54N, S:L455F, which were sequentially confirmed from the sample collected on Sep. 6th, are HK.3, a descendent lineage of EG.5, and FLip variants, which show growth advantage, were confirmed. Furthermore, the detection rate of FLip variants detected from Sep.6^th^ gradually increased from 17% to 67%.

**
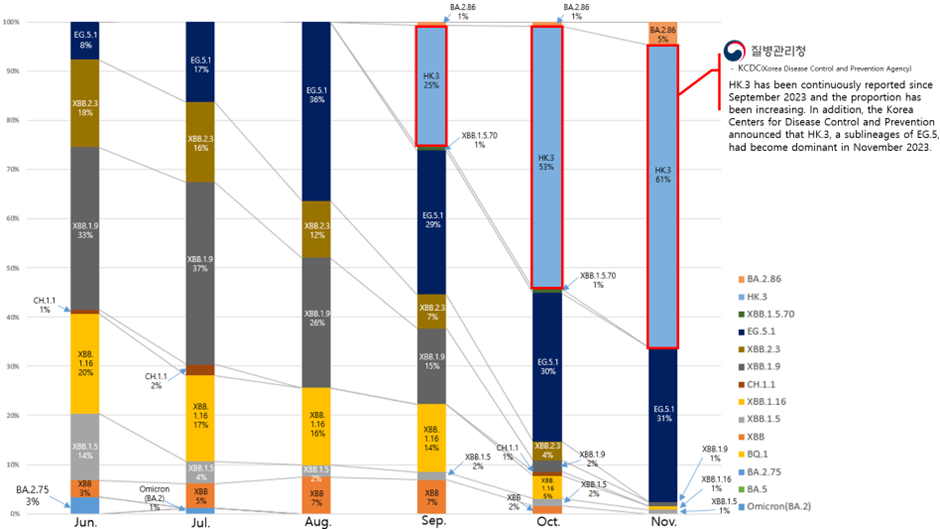
**

**Figure S3. Monthly COVID-19 Subvariants Trends & HK.3 dominance announcement by Korea Disease Control and Prevention Agency (KDCA).**

We confirmed the similarity between monthly detected mutations and changes in dominant species over time by comparing with ‘monthly COVID-19 mutation trends’ and ‘HK.3 dominance announcement by KDCA’. HK.3 has been continuously reported since Sep. 2023 and its proportion has been increasing. In addition, the KDCA announced that HK.3, a descendent lineage of EG.5, had become dominant in Nov. 2023 in a similar trend of its global prevalence.
